# Biomedical Applications of Biodegradable Polyesters

**DOI:** 10.3390/polym8010020

**Published:** 2016-01-16

**Authors:** Iman Manavitehrani, Ali Fathi, Hesham Badr, Sean Daly, Ali Negahi Shirazi, Fariba Dehghani

**Affiliations:** School of Chemical and Biomolecular Engineering, University of Sydney, NSW 2006, Australia; iman6901@uni.sydney.edu.au (I.M.); ali.fathi@sydney.edu.au (A.F.); hbad9313@uni.sydney.edu.au (H.B.); sean.daly@sydney.edu.au (S.D.); ali.negahi@sydney.edu.au (A.N.S.)

**Keywords:** polyesters, biodegradable, medical applications, tissue engineering

## Abstract

The focus in the field of biomedical engineering has shifted in recent years to biodegradable polymers and, in particular, polyesters. Dozens of polyester-based medical devices are commercially available, and every year more are introduced to the market. The mechanical performance and wide range of biodegradation properties of this class of polymers allow for high degrees of selectivity for targeted clinical applications. Recent research endeavors to expand the application of polymers have been driven by a need to target the general hydrophobic nature of polyesters and their limited cell motif sites. This review provides a comprehensive investigation into advanced strategies to modify polyesters and their clinical potential for future biomedical applications.

## 1. Introduction

The current market for regenerative implantation surgeries, therapeutic cell culturing and tissue repair is approximately US $23 billion, and it is anticipated to reach US $94.2 billion by the end of 2025 [1]. Synthetic biodegradable polyesters are considered the most commercially competitive polymers for these applications as they can be produced reproducibly in a cost-effective manner with a wide range of characteristics. Polyesters are also biocompatible, and biodegradable polymers are used for the manufacturing of different medical devices, such as sutures, plate, bone fixation devices, stent, screws and tissue repairs, as their physicochemical properties are suitable for a broad range of medical applications [2,3,4,5]. Polyesters are also used commercially in controlled drug delivery vehicles [6,7].

In all of the current commercial products, polyesters act as a biologically inert supporting material as a mesh or a drug-releasing vehicle. For more advanced medical and regenerative applications, polyesters are modified to tackle issues such as low cell adhesion, hydrophobicity, and inflammatory side-effects [8,9]. Consequently, the modification of polyesters has been one of the major research topics in the fields of material engineering and polymer science.

In this review, the properties of polyesters and the modification methods that have been implemented to improve some of the shortcomings of this class of polymers are discussed. Specifically, this review covers the applications and modifications of the most commonly used polyesters such as polylactic acid (PLA), poly(lactic-*co*-glycolic acid) (PLGA), poly(ε-caprolactone) (PCL), poly-3-hydroxybutyrate (or poly-β-hydroxybutyric acid, PHB), poly(3-hydroxybutyrate-*co*-3-hydroxyvalerate) (PHBV), poly(propylene carbonate) (PPC), poly(butylene succinate) (PBS) and poly(propylene fumarate) (PPF).

## 2. Synthesis of Polyesters

Polyesters are produced predominantly by using random polymerization, ring opening polymerization, and the block copolymerization techniques. For instance, PCL is produced by the ring opening polymerization of the ε-caprolactone using a catalyst such as an octoate [10]. The synthesis methods have been extensively reviewed in detail by many researchers; therefore, these synthesis approaches are not discussed in detail in this review [11,12,13,14,15]. The vast majority of the polyesters are derived from carbohydrate petroleum-based sources. Therefore, in recent decades, there has been a drive to find alternative sustainable polymers. Among all the polyesters, only PPC, PHB and PLA come from renewable sources.

PPC is produced in commercial scale from the ring opening reaction between CO_2_ and propylene oxide in the presence of an active catalyst such as zinc glutarate [16]. Similar ring opening polymerization mechanisms that are used to synthesise PPC and PCL are also used to synthesise PLA. The synthesis of PLA is a multi-step fermentation process starting with the biosynthesis of lactic acid. Lactic acid is then converted to its cyclic lactide foam and then polymerized via a metal catalyst [17,18].

PHB entirely is biosynthesized by an efficient fermentation process with different molecular weight (from 200 to 1500 kDa) using *diazotrophic* bacteria of *acetobacter* and *Rhizobium genus* [19]. PHB is primarily a product of carbon assimilation and it is employed by microorganisms as a form of energy storage molecules. The polycondensation of two molecules of acetyl-CoA leads to the formation of acetoacetyl-CoA that can be reduced to hydroxybutyric-CoA and polymerize PHB. However, the biosynthesis process of PHB is chirally selective and the resulting polymer typically has a polydispersity of around 2 or higher [20].

## 3. Properties of Polyesters

Linear aliphatic polyesters are mostly hydrophobic biodegradable polymers [21]. Their tunable physical and mechanical properties have extended their applications in the biomedical field [22]. It is easy to process these materials into desired structures with minimal risks of toxicity, immunogenicity, and infection. The main differentiating characteristics of polyesters are their mechanical performance and degradation behaviors that are discussed extensively as follows.

### 3.1. Mechanical Strength

In regenerative medicine, the mechanical property of a polymer plays a vital role in the selection of a biomaterial for any application. A robust biomaterial that does not mimic the mechanical strength of the targeted tissue interferes with the natural regeneration mechanism, and, ultimately, is a drawback for the damaged tissue repair [23]. The mechanical performance of bone, cartilage and cardiovascular tissues that are mostly treated with polyester-based implants are summarized in Table 1. In addition, this table outlines the mechanical performance of different polyesters and some medical devices. Medical devices such as screws and meshes are designed from polymers with the ultimate elongation strength of 200 MPa to fix cortical bones with the compression strength of 100–200 MPa.

There are numerous medical applications for polyester due to their broad range of mechanical properties. For instance, PGA has a relatively brittle structure as its ultimate strain is 30%. Therefore, PGA is not a desirable polyester for the fabrication of medical meshes as they are normally under high tensile strain. On the other hand, PPC displays a very flexible structure as its ultimate elongation at break is nearly 330%, which is at least five-fold higher than other polyesters. However, PPC may deform under elongation as this polymer displays very low tensile modulus, e.g., 22 MPa. Therefore, PPC is not a favorable candidate for the fabrication of medical screws, sutures, and meshes that are under constant tensile stress. PLGA and PLA posse significantly higher tensile modulus and strength compared to PPC. PLA displays the highest tensile stress (σ_m_= 55 MPa) and favorable ultimate elongation at breakage (ε_m_ = 30%–240%); hence, it has been broadly used for the fabrication of devices that are under constant tensile stress and high elongation.

**Table 1:** Mechanical properties of the biodegradable polyesters and a few tissues and commercially available biomaterials.

Material	Type	Tensile modulus (E, MPa)	Ultimate tensile strength (σ_m_, MPa)	Elongation at break (ε_m_, %)	Reference
**Tissues**	Bone (trabecular)	483	2	2.5	[24]
	Cartilage	10–100	10–40	15–20	[25]
	Cardiovascular	2–6	1	1200	[26]
**Medical devices**	Mg-based orthopaedic screw	Not reported	~200	~9	[27]
	Suture	~850	~37	~70	[28]
	Medical mesh (Vicryl^®^)	4.6 ± 0.6 (stiffness N/mm)	78.2 ± 10.5 (maximum force N/cm)	150 ± 6	[29]
**Polyesters**	PGA	7000–8400	890	30	[30]
	PLGA(50:50)	~2000	63.6	3–10	[31,32]
	PLA	3500	55	30–240	[33]
	PHB	3500	~40	5–8	[34]
	PPF	2000–3000	3–35	20.3	[22,35,36]
	PCL	~700	4–28	700–1000	[30,31]
	PPC	830	21.5	330	[37]
	PBS	~700	~17.5	~6	[38]

### 3.2. Degradation

An essential element in biomedical applications of polymers is the development of a temporary physical and mechanical support for the regeneration of newly formed tissues over time. Information about the degradation rate of a polymer is imperative for the design of various medical devices. For instance, a slow degradation rate of PLA provides the opportunity for the production of long-term orthopedic implants such as plates and screw [39,40,41]. However, PGA-based biomaterials are mainly used for the fabrication of sutures and drug delivery carriers due to their fast degradation [42,43]. Moreover, the rate of the degradation of polymers needs to be balanced to assure that the implanted device or the scaffold can provide the required mechanical strength for the regeneration of the newly formed tissue over time. For instance, in one case, a PLA-based implant, after an arthroscopic surgery, failed to regenerate the tissue and showed no signs of degradation, which resulted in some clinical complications for the patient [44].

The degradation is governed by different factors such as the nature of the polymer, composition, molecular weight, crystallinity, structure, thickness, surface properties and environmental conditions. The mechanical strength of a medical device or implant is also a function of degradation rate. For instance, molecular weight has a direct correlation with the rate of degradation, the higher molecular weight leads to slower degradation due to lengthy polymer chains [45]. However, the degree of crystallinity of some polyesters such as PLLA can proportionally affect the direct relationship between molecular weight and the degradation rate [46]. The indirect effect of crystallinity on the degradation rate is controversial as a few groups show that crystallinity of polyesters increases the degradation rate due to an increase in hydrophilicity [47,48]. In contrast, some groups display a slower rate with an increase in sample crystallinity [49].

The rate of degradation depends on the intrinsic chemical properties of polymers as well as the physical properties and the shape of the implant or device. The physical properties are important because the water diffusion and, consequently the hydrolysis of the polymer structures are affected by the contact surface area of the implants with the body fluids. Therefore, the degradation rates of different polyesters are reported within a range. Most of the polyesters are stable in the body for at least 12 months except PGA and its copolymer PLGA. This polymer has been copolymerized from LA and GA to acquire a relatively fast degradable polymer for medical applications. The degradation rate of PLGA can also be altered by changing the molar ratios of LA to GA. For instance, increasing the weight ratio of the GA to LA from 25:75 to 50:50 can accelerate the degradation by two-fold from 100 to 50 days.

Hydrolytic and enzymatic degradation are the primary mechanisms of degradation of polyesters through bulk- or surface degradation of implants [50]. Hydrolytic degradation has an autocatalytic nature and it proceeds through the hydrolysis of carboxylic groups of hydroxy acids [51], whereas the enzymatic degradation significantly depends on the enzyme that is responsible for the degradation of a specific molecule [52]. PCL, for instance, undergoes lipase-type enzymatic degradation in the presence of *Rhizopus delemer* lipase [53], *Rhizopus arrhizus* lipase, and *Pseudomonas* lipase [54]. Among these enzymes, *Pseudomonas* lipase significantly accelerates the process to totally degrade the highly crystalline PCL within four days [55], in contrast with hydrolytic degradation, which lasts several years. The general mechanism of degradation of polyesters is by bulk hydrolysis [56]. The presence of some enzymes may expedite the degradation of some of the polyesters. As a result of bulk degradation, there is a risk of a sudden loss in the structural stability of a polymeric structure.

It is critical to examine the biocompatibility and toxicity of any degradation product of a polymer for the design of biomedical devices. By-products of a bulk degradation of a polymer are released in the surrounding environment such as the host tissue. For instance, the release of acidic by-product from the degradation of PLA or PLGA may drop the pH of surrounding tissues and lead to cell necrosis and inflammation at the site [57,58,59]. It is therefore imperative to quantify the biodegradation products of polymers in order to study the biological behavior of the host environment upon the degradation of polymers systematically. The average logarithmic acid dissociation constant, p*K*_a_, of the intermediate degradation products of polyesters is used to quantify the acidity of the resulting products upon their degradation. The p*K*_a_ of the degradation products, the primary mechanisms of the degradation, and the *in vivo* degradation rate of the different polymers are summarized in Table 2.

**Table 2:** The degradation behavior of the biodegradable polyesters.

Polyesters	Degradation by-products (pKa)	*In vivo* degradation rate	Degradation mechanism
PLA (PLLA and PDLA)	Lactic acid (3.85) [60] (3.08) [61]	50% in 1–2 years [62] 98% in 12 months [63] 100% in >12 months [64] 100% in 12–16 month [31]	Hydrolysis through the action of enzymes [33]
PGA	Glycolic acid (3.83) [61,65]	100% in 2–3 months [62] 100% in 6–12 months [64]	Both enzymatic and non-enzymatic hydrolysis [62]
PLGA	Lactic acid (3.85)[60] (3.08) [61] Glycolic acid (3.83) [61,65]	100% in 100 days (75% LA: 25%GA) [66] 100% in 50–100 days [62]	Hydrolysis through the action of enzymes [31]
PPC	CO_2_ and Water (pathway and intermediates unknown)	6% in 200 days [67] No degradation after 2 months [68]	Hydrolysis, or enzyme mediation [69]
PHB	3-Hydroxybutyric acid (4.41 [70] or 4.7 [71])	35% degradation of molecular weight after 6 months [72] 60% degradation via thickness of pellet after 24 weeks [73]	Hydrolysis via nonspecific esterase enzymes [74,75]
PHBV	3-Hydroxybutyric acid (4.41 [70] or 4.7 [61,71]) 3-hydroxyvaleric acid (4.72 [61])	75% degradation via thickness of pellet after 24 weeks [73]	Hydrolysis via nonspecific esterase enzymes [74,75]
PBS	Succinic acid (4.21 and 5.64 for the first and second hydroxyl group) [76]	5–10 wt % in 100 days (*In vitro*) [76]	Enzymatic hydrolytic degradation [77]
PCL	Caproic acid (4.88) [78]	50% in 4 years [62] 1% in 6 months [79]	Hydrolytic degradation [79]
PPF	Fumaric acid (p*K*_a2_ = 4.44) [22]	Depends on the formulation and composition several months >24 [22]	Hydrolysis [80]

**Figure 1:**
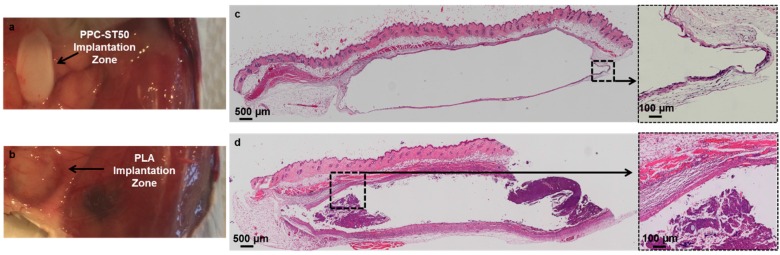
The explanation site of PPC-ST50 (**a**) and polylactic acid (PLA) (**b**) eight weeks post-surgery, and haematoxylin and eosin staining of paraffin sections of the implantation site at eight weeks around PPC-ST50 composite (**c**) and PLA (**d**). After eight weeks, a prominent foreign body reaction could be observed in the PLA implantation zone. However, the inflammatory response to the PPC-ST50 composite resolved dramatically. The PPC-ST50 and PLA scaffolds are present in the H&E images may not adhere to the glass slides during histological staining. Figure reproduced with permission from [81]. Copyright (2015) American Chemical Society.

Most of the polyesters, except PLA, PLGA, and PGA display a p*K*_a_ of 4–5, which is considered a relatively weak acidic environment, thus, the resulting biological inflammatory responses might not be severe. For instance, the haematoxylin and eosin staining results as displayed in Figure 1 shows that after eight weeks of PPC and PLA implantations in mice, there was no immune response to the PPC implant, whereas multi-layer fibrous tissues were noted around the PLA constructs due to the acidic degradation of this polymer. These results illustrate the favorable degradation properties of PPC [81]. Furthermore, it should be noted that the degradation byproducts of PHB can be useful for cell growth [82]. The average reported p*K*_a_ of the degradation products from PLA, PGA and PLGA are nearly 3.5, which can be considered as a semi-strong acidic environment. Therefore, upon clinical application of these polymers, care must be taken to ensure their long-term degradation.

### 3.3. Commercial Application of Polyesters

PLGA, PLA, and PCL are amongst the most widely used polyesters for the fabrication of sutures, drug delivery and implants as summarized in Table 3. PLGA has been used in commercial sutures since the 1970s (e.g., Vicryl^®^ with the latest and most widely used PGA-sutures on the market as Vicryl Rapide^®^ and Panacryl^®^, manufactured by Ethicon Inc., Edinburgh, United Kingdom) [83]. In addition, PLGA has been used for drug delivery applications, e.g., Lupron Depot^®^, Sandostatin^®^ Depot, and Risperdal^®^ Consta^®^ [83]. PCL is used for the fabrication of tissue repair patches (*i.e.*, Ethicon Inc., Edinburgh, United Kingdom) and as a filling agent to fill non-load bearing cavities in bone. PHB based biomaterials are mainly sutures (*i.e.*, Phantom Fiber™ (Tornier Co., Amsterdam, The Netherlands), MonoMax^®^ (Braun Surgical Co., Melsungen, Germany)) and surgical mesh such as TephaFlex^®^ mesh (Tepha Inc., Lexington, MA, USA), GalaFLEX mesh (Galatea Corp., Lexington, MA, USA) and Tornier^®^ surgical mesh (Tornier Co., Amsterdam, The Netherlands). Furthermore, a few medical disposable products are available in the market made of PBS such as Bionolle^®^ 1000 and 3000 (Showa Highpolymer Co. Ltd., Tokyo, Japan).

For load bearing applications, PLA is the most used polyester due to its intrinsic high mechanical strength (56.96 MPa compression and 3500 MPa tensile modulus) [33]. PLA is used in internal fixation devices, such as screws, plates, pins, and rods to support the repair of broken bones and hold them together [84]. However, *in vivo* studies show that PLA interferes with the bone remodeling process by imbalancing the number of osteoblast and osteoclasts during the bone remodeling [85,86]. Considering the commercially available polyester-based products as shown in Table 3, it can be observed that such products are mainly used as non-load bearing biomedical applications due to some unmet drawbacks. It is well-acknowledged that chemical and physical alterations of current-biodegradable polyesters are promising for enhancing their applications in the biomedical field. These approaches can be exploited to further extend the medical use of polyesters.

**Table 3:** Commercial products made from biodegradable polyesters and their applications.

Polymers	Applications	Commercial products
PLA	Fracture fixation [25], interference screws [25], suture anchors, meniscus repair [25], reconstructive surgeries [2], Vascular grafts [27], Adhesion Barriers [28], Articular cartilage repair [29], Bone graft substitute [2,30], Dural substitutes [2], Skin substitutes [2], Tissue augmentation [30], Scaffolds [8]	Proceed™ Surgical Mesh (Ethicon Inc.) , Artisorb™ Bioabsorbable GTR Barrier (Atrix laboratories, Fort Collins, CO, USA)
PLGA	(Composition 85:15): Interference screws [25], plates [25], suture anchors [25], Stents [38]/(Composition 50:50): Suture [25], drug delivery [25], Articular cartilage repair [39]/(Composition 90:10):Artificial skin [25], wound healing [25], hernia repair [2], suture [2], tissue engineered vascular grafts [2]	Rapidsorb^®^ plates (DePuy Synthes CMF, West Chester, PA,USA), Lactosorb^®^ TraumaPlatingSystem (Biomet, Inc., Warsaw, IN, USA) [l-lactide/glycolide = 82/18], RFS™ Screw System (Tornier), RFS™ (Resorbable Fixation System) Pin System (Tornier), Xinsorb BRS™ stent (Huaan Biotechnology Group, Gansu, China) REF1, Dermagraft^®^, Vicryl^®^ woven mesh (Ethicon Inc.) (Composition 90:10)
PCL	Suture coating [25], dental orthopedic implants [25], Tissue repair [2], hybrid tissue-engineered heart valves [2], Surgical meshes [2], cardiac patches [31], Vascular grafts [32], Adhesion Barriers [33], Dural substitutes [2], Stents [34], Ear implants [2], Tissue engineering scaffolds [16,35]	Tissue repair patches (Ethicon Inc.), Bulking and Filling agents (Angelo, 1996), DermaGraft™ (Organogenesis Inc., Canton, MD, USA)
PPF	Orthopedic implants [25], dental [25], foam coatings [25], drug delivery [25], Scaffolds [8,12]	-----
PPC	Scaffolds [87,88]	-----
PHB	Sutures (P4HB polymer) [2], screw fasteners for meniscal cartilage repair, Scaffold for tendon repair [2], Reconstructive surgeries (Surgical meshes) [2], Vascular grafts [32], Nerve repair [36,37], Bone tissue scaffold (P3HB) [26], Wound dressing (P3HB) [2], hemostats (P4HB) [2], Stents [38]	Phantom Fiber™ suture (Tornier Co.), MonoMax^®^ suture (Braun Surgical Co.), BioFiber™ scaffold (P4HB polymer) (Tornier Co.), TephaFlex^®^ mesh (Tepha Inc.) (P4HB polymer), GalaFLEX mesh (Galatea Corp.), Tornier^®^ surgical mesh (Tornier Co.)
PHBV	Scaffolds [89,90]	-----
PBS	Stents [2], Sterilization wrap [2], Diagnostic or Therapeutic Imaging	Disposable Medical Products-Bionolle^®^ 1000 and 3000 (Showa Highpolymer Co. Ltd.)

## 4. Modification of Polyesters

Polyesters are broadly used for biomedical applications. However, different approaches are undertaken to address their shortcomings. Polyesters are commonly hydrophobic with a low number of cell-motif sites within their structures which results in inferior cell interaction behavior. Different physical and chemical modification techniques have been used to enhance their biological activities that are briefly described in this section.

In the physical modification, the molecular structure of polymers is not changed and an additional component(s) is mixed with the polymer; either by solvent casting or melt blending techniques. In the chemical modification, the molecular structure of the polymer is changed. There are two pathways; (a) copolymerization of the building blocks of polyesters to form a new class of polymers; and (b) modification of the polymer chain of the polyesters post-synthesis. In the following sections, the physical and chemical modification methods of the most used biodegradable polyesters for biomedical applications are discussed.

### 4.1. PLA

According to the European Bioplastics Association, more than 142,000 tons of PLA was consumed in 2013 which is more than 11.4% of the global bioplastic production capacity [91]. In biomedical applications, this polymer is also the most commonly used, and, thus, has been extensively modified by incorporating different organic and inorganic components. Additionally, PLA is the only member of the polyester family that has been used for load bearing applications such as orthopedic screws and plates, owing to the high mechanical strength of this polymer [92,93]. The properties of PLA depend on its molecular characteristics, crystallinity, morphology and degree of chain orientation.

Lactic acid, the building monomer of PLA, provides chiral configuration for PLA including D and l-polylactic acid. For load bearing applications, l-PLA is preferable because of the high strength and toughness of the resulting polymer; however, d-PLA is used in drug delivery systems due to its faster degradation rate. Three different crystallinity of the PLA including α, β, and γ forms are available. These three crystalline structures of PLA (α, β, and γ forms) display melting points of 185, 175 and 235 °C, respectively [94]. Regardless of the crystalline structure, and chiral configurations, PLA exhibits a very hydrophobic nature and a low ultimate elongation strain of nearly 10% [95]. In addition, PLA degradation in the body decreases the pH of surrounding tissues substantially, which may cause clinical complications such as necrosis and delayed healing. Similar to all other polyesters, the lack of cell motif sites within the structure of this polymer has also been a significant driving force to modify PLA. Therefore, PLA has been changed (a) to enhance its hydrophilic properties; (b) to increase the ultimate elongation strain; (c) to address the formation of acidic biodegradation products; (d) to improve the bioactivity; (e) and to increase the number of cell motif sites within its structure. Table 4 summarizes some of these physical and chemical modification approaches.

**Table 4:** Polylactic acid (PLA)-based structures applied in biomedical and tissue engineering applications.

Polyester	Modifier	Concentration (wt %)	Porosity (%)	Mechanical properties (MPa)	Enhanced properties	Reference
PLA	PU	50	79	80 (C-M)	Mechanical performances	[96]
	PCL	50	81.5 ± 1.2	0.3 (C-S)		[97]
	PEG	20	86.75	1830 (Y-M) (nano-indentation method)		[98]
	Triclosan	20	Solid structure	61.98 ± 0.3 (T-S)	Cell binding	[99]
	Chitosan and keratin	30% chitosan and 4% keratin	Solid structure	35 (T-S)		[100]
	BG	40	0.211 (cm^3^/g)	0.3 (C-S)	Bioactivity and neutralize the acidic degradation	[101]
	Carbonated apatite	30	70	2.2 (R)		[102]
	HA	50	85	857 ± 0.268 (E-M)		[103]
	Calcium phosphate	50	96.58 ± 0.85	0.147 ± 0.02 (S)		[104]
	Halloysite nanotube	10	Solid fibers	10.4 (T-M)		[105]
PLGA	PHBV	50	81.273 ± 2.192	1.5 (C-M)	Mechanical performances	[106]
	Gelatin	30	78.41	6.43 ± 0.37 (T-S)	Hydrophilicity	[107]
	Nano HA	5	89.3 ± 1.4	1.3546 ± 0.053 (C-M)	Bioactivity	[108]
	BG	1	93 ± 2	0.412 ± 0.057 (C-S)		[109]
	Silica nanoparticles	10	Solid fibers	114 ± 18.6 (Y-M)		[110]

Y-M: Young’s modulus; T-S: Tensile strength; C-S: compressive strength; R: resistance; E-M: Elastic modulus; S: stiffness; T-M: Tensile modulus; C-M: Compressive modulus.

The primary motivation to chemically modify PLA and to copolymerize lactic acid with glycolic acid to form PLGA was to develop a polymer with a more hydrophilic nature that degrades into less acidic products. This concept was initially hypothesized as glycolic acid has higher (more neutral) p*K*_a_ compared with lactic acid. However, the degradation products of PLGA are lactic acid and glycolic acid, and both of them still lower the pH of the surrounding tissue. In addition, PLGA displays a faster degradation rate, which is favorable for biomedical applications such as bioabsorbable sutures or drug delivery devices. Therefore, in parallel with PLA, the medical use of PLGA has also been expanded and, thus, a wide range of physical and chemical modifications have been made to both PLA and PLGA to enhance their properties.

The mechanical properties of PLA are favorable for load bearing applications, and the only mechanical shortcoming of PLA is its low ultimate tensile strain (e.g., around 10%). To enhance this property of PLA, thermoplastic polyurethane (TPU) and PCL have been physically added to this polymer [96,97]. TPU can tune its tensile modulus within the range of 7–1007 MPa at the strain of above 15% for neat PLA and a blend with 1:1 weight ratio, respectively. While, the addition of 50 wt %, PCL increases the elongation at break by nearly 10 fold (107% ± 4.7%). PLGA intrinsically displays very stretchable behavior with high ultimate tensile strain. However, the elongation and compression moduli of this polymer are lower than PLA, which drives the use of PLA for load bearing applications. In few cases, PLGA is blended with other polymers such as PHBV, which is a brittle but stiff polymer (high tensile modulus), to enhance the compression modulus and tensile moduli by two to three fold [106].

For tissue regeneration applications, the cell interaction behavior of PLA and PLGA-based composites needs to be improved, and the first material of choice to address this challenge is natural polymers, such as polysaccharides, polypeptides, and proteins. Tanase *et al.* introduced a polyester blend modified with chitosan and keratin to enhance cell interactions of the polyester [100]. An *in vitro* cell study using human osteosarcoma cell line shows a good cell viability and proliferation. Furthermore, the incorporation of polyethylene glycol (PEG) into the PLA matrix is used to enhance the surface hydrophilicity, and therefore, its biological behavior [98]. However, the addition of PEG results in a decrease in mechanical performance.

The cell interaction of PLGA also needs to be improved. Similar to PLA, natural polymers have been widely used to enhance the cell interaction capability of PLGA. Accordingly, PLGA knitted mesh is modified with collagen type I to develop a supporting biomaterial for cartilage and bone regeneration applications [111,112]. For chondrocyte growth and proliferation to help cartilage repair, 3D biodegradable scaffolds were formed with a different configuration of collagen inside the PLGA matrix and led to homogeneous cell distribution, natural chondrocyte morphology, and abundant cartilaginous ECM deposition. However, the mechanical strength of the most promising scaffold was at least half of the requirement for cartilage regeneration [111]. In another study, laminated mesh of PLGA and collagen was modified this time for bone-cartilage interface reconstruction. In this study, the collagen microsponge was crosslinked by treatment with 25% glutaraldehyde saturated vapor to cover the surface of the PLGA knitted mesh. The tissue engineered scaffold possessed the same behavior as a native osteochondral plug nine weeks after post-implantation regarding DNA expression of collagen type I and II. Another research group modified the surface of PLGA with poly-l-lysine using a water-in-oil-in-water emulsion or solvent evaporation technique [113]. Surface modification promoted the cell differentiation; however, it showed an adverse effect on the mechanical properties of PLGA. Gelatin was also used to modify a biodegradable polyester microfiber using electrospinning [107]. These examples demonstrate that various strategies can be used to enhance the biological properties of PLA and PLGA by incorporating natural polymers. The addition of natural proteins and polysaccharides, however, cannot potentially address the acidic degradation products and low bioactivity of PLA. To tackle this problem and to enhance the bioactivity of the PLA and PLGA based constructs, bioactive ceramics can be added to PLA, as the degradation products of ceramics are mostly basic and can promote the proliferation of native bones in the load bearing applications of these polymers.

There are numerous studies as summarized in Table 4 that investigates the effect of adding bioactive ceramics such as hydroxyapatite (HA) and β-tricalcium phosphate (β-TCP) to neutralize the acidic degradation media of polyesters and to evoke bioactive properties to these polymers [57,114]. The results of these studies demonstrate that the basic degradation of ceramic particles can neutralize the acidic environment. In a more clinical-based study, a method is developed for the treatment of skull defects by using PLA plates supplemented with carbonated apatite bone cement [115]. In these implantable plates, carbonated apatite cement particles are dispersed into the PLA sheets and are fixed to skull fractures. After 3–60 months’ follow-up, no complications concerning dislodgement or structural failure of the cranioplasty construct were observed. Several studies reported the positive impact of adding bone cement particles within the structure of PLA to enhance the cell interaction and bioactivity of PLA based structures [116,117]. Care must be taken to prepare a homogeneous composite of ceramic-polymer to achieve suitable mechanical properties and also predictable degradation behavior.

Hydrolysis by an alkali is the first step of chemical modification to provide an active site on the surface of a polymer [118]. In this procedure, the ester bond of biodegradable polymer is activated to bond with the hydrophilic –COOH and –OH or reactive –NH_2_ groups in components such as an arginine-glycine-aspartic acid (RGD)-containing peptides, chitosan (CS), arginine and lysine, PEG, collagen, *etc*. Enhancement of wettability of the surface and biocompatibility of the scaffold are the main aims of these surface modifications. For instance, a PLA modified with RGD results in improvement in the cell densities and proliferation mediated through RGD–integrin interactions [119]. In spite of all the mentioned advantageous features for the polymers driven by post-polymerization, the possibility of side reactions, such as chain scission and racemization along with the complexity of this process, are the main disadvantages of this method. Therefore, post-polymerization functionalization is not the preferred route to obtain functional polyesters, and, also, these methods are not practical for the formation of 3D structures [21].

Advanced chemical modification methods are carried out to improve the physical and biological characteristics of both PLA and PLGA for the fabrication of 3D structures [21]. A general synthetic route for functionalization of PLA is copolymerization with 3-(*S*)-[(benzyloxycarbonyl)methyl]-1,4- dioxane-2,5-dione protected with benzyl alcohol followed by diazotization with sodium nitrite [120]. The deprotection process performed via catalytic hydrogenolysis of the benzyl groups using both PtO_2_ and Pd/C catalysts results in an enhanced *in vitro* hydrolysis rate compared to PLA. The monomer functionalization has been extensively studied; however, few types of research evaluated the monomer functionalized polyesters for tissue engineering applications due to unknown biological properties that may lead to clinical complications [121,122,123,124].

The ring opening copolymerization of lactic acid through its carboxyl and hydroxyl groups is a possible way to chemically modify PLA and can produce high molecular weight polymers in combination with glycolide, δ-valerolactone, and trimethylene carbonate, as well as with monomers like ethylene oxide [125]. For instance, for drug delivery application, a range of PLA-PEG copolymers have been synthesized by using PEG block with a certain molecular weight and varying PLA segment lengths (e.g., *M*_n_ = 2000–110,000) using ring-opening polymerization of d,l-lactide catalyzed by stannous octoate [126]. Furthermore, PLA copolymerized with polyurethanes by copolymerization of l-LA and 1,4-butanediol to acquire mechanical properties for soft tissue engineering [127]. In addition to these general approaches to enhancing the physical and biological properties of PLA-based materials, more advanced polymer synthesis methods have been employed to make more clinically appropriate PLA-based materials. For instance, to eradicate the need for using organic solvents, there are numerous studies that attempt to generate water-soluble forms of PLA by grafting different molecules to this polyester.

**Figure 2:**
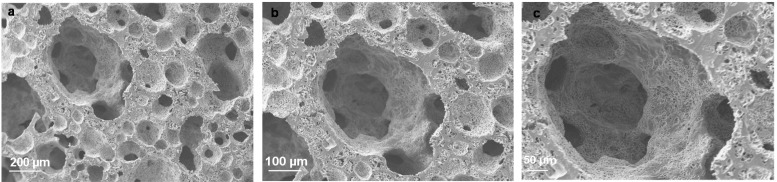
The micro and macroporous structure of PLEOF-methacrylated gelatin interpenetrated network. Figure reproduced from [138], with permission from Elsevier.

Polymer grafting such as chitosan-grafted-PLA can be prepared by attaching PLA to the chitosan main chain, and these materials can be dissolved in low pH aqueous based solution [128,129]. PLA and PEG were also functionalized with FuCl to form a water soluble and crosslinkable form of PLA. This polymer has been extensively studied and analyzed by Jabbari’s research group [130,131,132,133,134]. In yet another study, a green approach was developed to synthesize this polymer under high-pressure CO_2_ to eradicate even the use of organic solvent during its synthesis [135]. Conducting the synthesis in CO_2_ gas expanded solution remarkably increased the fumarate crosslinking active site in the backbone of poly(lactide-ethylene oxide fumarate) (PLEOF) copolymer, hence, enhancing the mechanical properties and osteoblast cell adhesion and proliferation [135,136]. Interpenetrated polymer networks of PLEOF reinforced with gelatin and methacrylated gelatin were also synthesized with enhanced primary human osteoblast cell adhesion and proliferation [137,138]. As shown in Figure 2, these interpenetrating polymer network structures were composed of micro (~20 μm), and macropores (540 μm) pores that promote the nutrient mass transfer and cell growth, respectively.

To form injectable hydrogels for various medical applications, we further chemically modify PLA [139]. In this approach, we copolymerized PLA with hydroxyethyl methacrylate (HEMA) with a ring-opening polymerization technique. The resulting PLA/HEMA was then conjugated with a number of monomers, e.g., NIPAAM, NAS, and OEGMA to form water soluble, temperature responsive and protein reactive molecules. These polymers can be used for cartilage and bone regeneration applications [140,141,142]. All these chemical modification approaches demonstrate the polyesters are modifiable and their properties can be tuned for a broad range of medical applications.

### 4.2. PHA Family

Polyhydroxyalkanoates (PHAs) are synthetic biodegradable polyesters that can be biosynthesized with the fermentation of microorganism, and can also be chemically synthesized [143]. PHA is produced by the biosynthesis pathway through acetyl-CoA which leads to the production of PHB [144]. PHB and PHBV are the most thoroughly studied forms of the PHA family for biomedical applications due to their biocompatibility, biodegradability, and adjustable mechanical properties. The biodegradation of PHB and other PHA derivatives are driven by hydrolysis of the ester bond [74,75]. Their degradation products, such as a β-hydroxybutyric acid (3HB) and 3-hydroxyvaleric acid, are less acidic than lactic and glycolic acid with pK_a_ values of 4.7 [71] and 4.72 [61], respectively. The mechanisms of PHB degradation are thermal, enzymatic or hydrolytic. Hydrolytic degradation of PHB releases 3HB, which is a normal metabolite in human blood; therefore, in the absence of endotoxin, the biodegradation of PHB produced by bacteria does not cause any physiological reaction. Moreover, 3HB by itself has pharmaceutical and biomedical applications as its derivatives decrease cell apoptosis [61,145]. This property provides a unique feature for regeneration and drug delivery applications of PHB and other polymers in the PHA family.

Propionate, valerate, hexanoate, and 1,4-butanediol can be added to produce random copolymers and block polymers, such as poly(3-hydroxybutyrate-*co*-3-hydropropionate), poly(3-hydroxybutyrate-*co*-3-hydroxyvalerate) (PHBV), poly(3-hydroxybutyrate-*co*-3-hydroxyhexanoate), and poly(3-hydroxybutyrate-*co*-4-hydroxybutyrate) [144,146]. Poly(3-hydroxybutyrate-*co*-3-hydroxyhexanoate) is another member of PHA family that is physically blended with PHB. The main limiting factors for the medical applications of the PHA family are (a) low ultimate tensile strain (b) minimal cell interaction capacity. To tackle these shortcomings, these polymers have been combined with numerous other natural and synthetic polymers. Table 5 summarizes some of the modifications that have been carried out on PHB and PHBV.

**Table 5:** The physicochemical modifications of the polyhydroxyalkanoates (PHA)-based polyesters in the field of biomedical and tissue engineering.

Polyester	Modifier	Concentration (wt %)	Porosity (%)	Mechanical properties (MPa)	Enhanced properties	Reference
PHB	HA	30	Solid film	1400 (S-M)	Bioactivity	[147]
	Herafill	30	Solid film	2800 (Y-M)		[148]
	BG	10	85	Not reported		[149]
PHBV	Chitin	10	Not reported	7.12 ± 0.24 (C-M)	Cell binding	[89]
	Silk and nHA	5 (*w*/*v*) %	71.44 ± 0.81	0.72 ± 0.26 (Y-M (kPa))	Bioactivity	[150]
	Calcium silicate	20	80	~ 33 ^1^ (C-M)		[151]
	HA	10	Solid fibers	4.19 ± 0.19 (U-S)		[152]

C-M: Compressive modulus, Y-M: Young’s modulus, S-M: storage modulus, T-S: Tensile strength; 1. After 12 weeks implantation.

Chitosan, chitin, and chondroitin sulfate are used to improve the biological and mechanical elongation properties of the PHA family [89,90]. For instance, after adding 10 wt % of chitin nanocrystals, the compressive modulus of PHA increases by 28% from 5.21 ± 0.14 MPa to 7.12 ± 0.24 MPa. The different weight ratio of PEO (polyethylene oxide) is also used to improve the tensile strength and the elongation at break of PHB [153]. The results showed that the addition of 10 wt % PEO improves the tensile strength by 40% while maintaining the elongation at break at a constant value; however, adding 50 wt % PEO causes a 69% decrease in the tensile strength while increasing the elongation at break significantly. Therefore, PHB blend exhibits more elastic properties with lower toughness in comparison with PHB homopolymer.

Nano-HA, bioactive glass, tricalcium phosphate, calcium silicate, zirconium dioxide and herafill^®^ are some examples of inorganic compounds that have been added to PHB and PHBV to increase their bioactivity and cell interaction capacity for bone implants and tissue engineering [148,149,150,151,152,154,155,156,157]. For instance, the addition of 20 wt % calcium silicates enhances the cell adhesion, distribution and proliferation and bone-bioactivity of the composite. Furthermore, the introduction of micro and nanoparticles of 45S5 Bioglass grades, to interconnect a highly porous PHB with 85% porosity, results in the formation of a HA layer with a Ca/P ratio of 1.57 after 10 days of being immersed in SBF. This rapid formation of HA within this short period reveals that the fabricated composite is highly bioactive and favorable for bone regeneration applications. However, the pH of the degradation media increased to 8.5 after the addition of 10 wt % nano BG particles due to the basic degradation of ceramics that may lead to some clinical complications.

The chemical modification of PHB via either graft copolymerization or *in situ* polymerization or multi-block copolymerization was also studied [158]. To this end, the hydroxyl end group of PEG is first functionalized with acryloyl chloride to form PEGM (polyethylene glycol methacrylate). Then, the free radical copolymerization of acrylates groups of PEGM under UV irradiation takes place in chloroform. The resulted copolymer was shown to possess significantly higher equilibrium water content that may lead to a more hydrophilic structure than that of PHB, which is vital for cell interaction in biomedical applications.

The full potential of PHB for tissue engineering and drug delivery applications has not yet been exploited. This is because, the mixing of PHB with other polymers is technically challenging: PHB is soluble in very few solvents, *i.e.*, chloroform, dichloromethane, and dimethyl formamide, which is a hindrance for the solvent casting method and the formation of composite structures. In addition, thermal molding is also challenging, as above 150 °C most of the PHA based polymers break down to fatally toxic *trans*-crotonic acids. Addressing these challenges may open up an avenue for further modification of PHA polymers and their future medical applications.

The exceptional stereochemical regularity of PHB that leads to a high degree of crystallinity in the range of 60%–80% is another limiting factor for the biomedical application of PHB [159]. This highly crystalline structure along with tacticity is the main material characteristics of PHB that affects the processability of PHB. Chemical modification of this biodegradable polyester such as multi-block copolymerization with PEG can decrease the degree of crystallinity of PHB and extend the applications of this polymer in the biomedical field [160].

### 4.3. PPC

PPC is a biodegradable aliphatic polyester that was first synthesized by the copolymerization of carbon dioxide (CO_2_) and propylene oxide at the end of the 1960s [161]. PPC is an amorphous biodegradable polyester, and its thermal properties such as thermal decomposition, melting temperature and glass transition temperature are in the range of 240–260 °C, 150–170 °C and 37–42 °C, respectively [69,162,163]. Comparable thermal, mechanical, biocompatibility and degradation properties of PPC with other aliphatic polyesters, which have been broadly used in tissue engineering, motivate researchers to investigate the feasibility of using PPC as a biomaterial [87,164,165,166,167]. The final degradation products of PPC are CO_2_, and water, which could solve the issue of inflammation that commonly occurs during the degradation of other polyesters. The biodegradation mechanism of PPC, e.g., the nature of the resulting intermediate substances, is not clearly understood [164].

The first biocompatibility of PPC was proved by Kavaguchi *et al.* at 1983 [165]. The results demonstrated that PPC is a biocompatible polyester because there was no inflammatory response and retardation in animals leads to weight gain. In addition, the degradation of PPC has been studied for its use as a surgical polymer, or as a slow-release substrate in the peritoneal cavity in rats. As a consequence of the small surface area of pellets that were implanted in rats, the degradation of PPC was negligible within two months. Another study by Kim *et al.* [164] focused on evaluating the biodegradation of PPC. Three different mechanisms including oxidative degradation, hydrolytic degradation, and enzymatic degradation have been proposed, but enzymatic degradation has been selected as the primary process. The cell attachment on PPC is very limited due to its highly hydrophobic nature. Therefore, PPC is physically and chemically modified for biomedical applications. The effect of some modification processes is summarized in Table 6.

The surface hydrophilicity of PPC based constructs has been enhanced by using well-established surface modification techniques such as UV irradiation and plasma coating [167,168]. Low-power deep UV radiations were used to enhance the cell attachment and proliferation on the surface of electrospun PPC [167]. This surface treatment led to a higher adsorption of the protein layer followed by an improvement in cell attachment. Oxygen plasma treatment method was also used to enhance the wettability of PPC based constructs. To this end, parallel-aligned PPC microfibers with a fiber diameter of 1.48 ± 0.42 µm were prepared firstly; then, chitosan nanofibers with a fiber diameter size of 278 ± 98 nm were introduced into the PPC fiber mats by freeze drying. Oxygen plasma treatment at a pressure of 0.025 mtorr and radio power generating oxygen plasma 100 W was used. The surface modification resulted in the fall of water contact angle from 122.3° ± 0.4° for neat PPC scaffolds to 53.8° ± 1.6° for plasma treated samples. However, it should be noted that the initial reported contact angle data for neat PPC conflicts with other literature, which have reported an average of 76° [164,169]. The cell attachment, proliferation, and cell–scaffold interactions were enhanced in PPC microfibers and chitosan nanofibers.

**Table 6:** Organic and inorganic components added to the poly(propylene carbonate) (PPC) matrices.

Polyester	Modifier	Concentration (wt %)	Porosity (%)	Mechanical properties (MPa)	Enhanced properties	Reference
PPC	Chitosan	5	91.9	14.2 ± 0.56 (C-M)	Hydrophilicity and cell binding	[87]
	Chitosan	7	Solid fibers	5.0 ± 0.8 (T-S)		[168]
	PEI and Gelatin	Coating	92.3	0.4 (C-M)		[166,169]
	Graphene oxide	1	83.54	1 (C-M)	Physical characteristics such as mechanical performances and porosity	[170]
	Gelatin	15	Solid fibers	2.88 ± 0.82 (T-S)		[88]
	Starch	50	Solid disk	33.9 (C-M)		[81]

C-M: Compressive modulus; T-S: Tensile strength.

For the fabrication of 3D structures with more favorable hydrophilic properties and cell behavior characteristics, PPC is mixed with other natural polymers. A composite of PPC and gelatin, in trifluoroethanol as a solvent and at low mass content of gelatin, with improved wettability and hydrophilicity was produced by Jing *et al.* [88]. Gelatin was used in this study to improve the cell attachment and proliferation of scaffolds; however, phase separation occurred when the mass content of gelatin was higher than 5% due to the usage of immiscible solvent. The phase separation resulted in the formation of a non-uniform fibrous structure and large splash defects. The study shows that the PPC/gelatin composite scaffolds exhibit better performance in the wettability and mechanical tests as well as cell culture experiments when compared to those of pure PPC frameworks. On the same topic, to address the phase separation challenge, micro- and nano-fibers of PPC and chitosan were separately generated and mixed subsequently [168]. The miscibility of graphite within the structure of PPC was also challenging. Graphite with an average size of 7.4 μm and a nanometer-sized thickness of 30–50 nm was used to improve the physical properties of PPC [171]. This research revealed that poor dispersion occurs in composite films with high graphite content, and the maximum value of 2 wt % graphite shows better morphological structures, thermal properties, mechanical properties and barrier properties. Another study investigates the usage of graphene oxide (GO) to fill PPC matrix to enhance its mechanical performance [172]. The dispersion of the filler within the structure of PPC was also technically challenging.

GO-PPC composite preparation was carried out in solution phase; while a certain amount of GO/H_2_O solution was added to the PPC/tetra hydro furan solution. To this end, syringe titration was used to avoid coagulation of PPC in water. Toughening PPC with rubbery non-isocyanate polyurethane (NIPU) was also considered [173]. The equilibrium between self-associating hydrogen bonding and intermolecular interaction formed between PPC and NIPU was shown to affect the miscibility and the morphology of the blends. Moreover, the study showed that the addition of 10 wt % of NIPU leads to a three-fold increase of impact strength in comparison to neat PPC. However, when the NIPU loading reached 13 wt %, NIPU agglomerated in the matrix leading a decline in toughness.

Using the solvent casting method for the modification and processing of PPC based construct is challenging. This is because, similar to PHA based families, PPC is only soluble in few solvents such as dichloromethane and tetrahydrofuran [69]. The use of a thermal blending method, therefore, is deemed to be the most convenient way to form composite structures. This melt blending process has been widely used to produce a PPC-polysaccharide blend for packaging purposes [174,175,176,177]. More recently, it has been shown that a composite of PPC and starch can be produced via a melt blending method that enhances the physical characteristics of polyester and eradicates the miscibility issue [81]. However, the starch microparticles that are embedded into the PPC matrix were thoroughly covered by the hydrophobic PPC. A new emerging strategy to increase the hydrophilicity of the polyesters is the usage of plasticizers such as glycerol and sorbitol [178]. This problem was alleviated by the addition of plasticizers such as glycerol and water during PPC and thermoplastic starch blending [179]. This innovation led to the fabrication of a biodegradable plastic bag without using any cytotoxic plasticizer, which could have implications for future biomedical applications.

### 4.4. PBS

The poly(alkaline dicarboxylate) family of polymers are biodegradable polyesters. PBS is the most commonly used polymer in this family of polymers due to its relatively low production cost, good thermal and mechanical properties, and ease of processability [180,181]. The primary degradation product of PBS is succinic acid that is an intermediate of the tricarboxylic acid cycle or Krebs cycle; thus, it degrades inside the body with final products of water and carbon dioxide [182]. An important factor that limits the application of PBS in the biomedical field is its hydrophobicity with the reported contact angle of 75.03 ± 0.38 that causes little cell interaction [183]. Composites of PBS with different hydrophilic polymers were formed to enhance the wettability and potentially the biological properties of the polyester [184,185,186].

An electrospun composite microfiber of PBS and PEG was developed for tissue regeneration. The primary intention in order to blend these two polymers was to use PEG as a porogen by leaching it in an aqueous solution. However, the complete removal of the porogen was not feasible due to the low porosity of the fabricated structure, leading to the formation of a composite semi-porous PBS/PEG structure. The composite displayed more hydrophilic properties, but the cell interaction capacity of the polymer was limited, as neither of the polymers had any cell motif sites [186]. The melt blends of PBS and chitosan scaffolds with a 50 wt % filler have been used for cartilage and bone tissue engineering by multiple research groups [182,184,185]. The solubility of PBS and chitosan in acidic aqueous solutions allows for the formation of one phase solution and, thus, the formation of composite structures. The PBS/chitosan biodegradable scaffold supported the osteogenic differentiation of human bone mesenchymal stem cells cultured on their surface *in vitro*. The culture media was supplemented with osteogenic additives. Results from this study, therefore, cannot fully confirm the osteogenic nature of the PBS/chitosan. Another *in vivo* study in nude mice validates bone growth at the site of the cranial defect by implanting PBS/chitosan scaffolds with pre-cultured mesenchymal stem cells. The microCT analysis shows that the bone healing process began eight weeks post-implantation. This result is not very promising as bone regeneration after eight weeks is common in normal healing processes. Additionally, the Western blot assay reveals that the bone marrow-derived mesenchymal progenitor cell line cultured on the scaffold was being differentiated toward the chondrogenic pathway for periods of up to three weeks [182].

Chitin and chondroitin sulfate nanoparticle are added to the PBS to improve the cell motif of the biodegradable polyester to provide cell adhesion for skin tissue engineering [187]. Human dermal fibroblast cells adhered and proliferated on the surface of the scaffold and proved the suitability of the constructs for skin regeneration. Live-dead assay of the cells on the surface of the composite structure exhibits a significant improvement in cell viability due to the acceleration of wound healing because of the enhancement of the influx of fibroblasts into the wound, the increase of proteoglycan synthesis and collagen-II and also the exertion of anti-inflammatory activity. To fabricate PBS based composites for bone regeneration applications, HA particles are added to PBS films. To this end, a biomimetic method that involved the formation of HA layer on the PBS ionomer inside SBF was used. [188]. In this novel approach, sodium sulfonate ionic groups with negative charges were found to lead to the binding of plenty of the Ca^2+^ ions on the surface of PBS and form a stable layer of HA, which is favorable for the ingrowth of the surrounding tissue and bone formation. Furthermore, 20 wt % β-tricalcium phosphates (TCP) were added to the PBS to possess *in vitro* osteoblast growth and differentiation [189]. Results revealed that the incorporation of calcium phosphate not only improves the bioactivity of the scaffold but also increases the wettability of the films by 23.89% that is satisfactory for cell ingrowth.

Different chemical and physical modification approaches have been carried out on PBS to increase the hydrophilicity and the biological properties of this polymer. However, the most prominent drawback for the clinical application of this polymer is its brittle nature. As an illustration, PBS has the lowest ultimate elongation strain (6%) with one of the lowest ultimate tensile strengths (17 MPa) among all polyesters. To the best of our knowledge, there is no research that endeavors to improve the stretchability of this polymer. Addressing this important drawback of PBS may expand the application of this polymer in biomedicine and tissue regeneration.

### 4.5. PCL

Poly (ε-caprolactone) is an aliphatic polyester that has been widely considered for biomedical applications including drug delivery and tissue engineering [190]. Its compatibility with a broad range of drugs enables uniform drug distribution in the formulation matrix, and its long-term degradation facilitates drug release up to several months [191]. The homopolymer PCL has a total degradation of two to four years (depending on the starting molecular weight of the polymer) with hydrolysis as the primary degradation mechanism [10]. Pitt *et al.* showed that the mechanism of *in vivo* degradation of PCL, PLA, and their random copolymers was qualitatively the same [10]. PCL was studied extensively for tissue engineering applications, such as scaffold for bone tissue engineering, and other advanced 3D prototype blend composites for hard tissue engineering [192]. Among PCL’s commercial applications, a monofilament suture, MONOCRYLs^®^, which is made of a PCL-Glycolide copolymer and a contraceptive product, Capronor^®^, which can deliver a drug for over a year, has been commercially available for over 25 years [83]. PCL is modified to enhance the cell binding capacity, to increase its compression and tensile strength and also to accelerate the degradation rate of this polyester. Some modification approaches to PCL are summarized in Table 7.

**Table 7:** Modification methods of poly (ε-caprolactone) (PCL)-based composites for biomedical and tissue engineering applications.

Polyester	Modifier	Concentration (wt %)	Porosity (%)	Mechanical properties (MPa)	Enhanced properties	Reference
PCL	Chitosan	25	Solid fibers	1.78 ± 0.25 (T-S)	Hydrophilicity and cell binding	[193]
	Collagen	Coating	93.9 ± 0.4	5 (Y-M)		[194]
	Gelatin and Collagen	20% gelatin and 1.5% collagen	Solid fibers	1.29 (T-S)		[195]
	Elastin	30	91	1.30 ± 0.07 (C-M)		
	Alginate	5	92	0.72 ± 0.04 (T-S)		[196]
	Nanofiber PLA	10	79.7	Not reported	Physical characteristics such as mechanical properties and porosity	[197]
	MWNTs	2	Solid disk	110 (T-M)		[198]
	Phlorotannin nanofibers	5	Solid fibers	57.8 ± 6.6 (Y-M)		[199]
	Silica	5.4	63.3 ± 2.0	13.6 ± 1.6 (Y-M)	Degradation behavior and bioactivity	[200]
	BG	21 vol %	0.1 (cm^3^/g)	1310 (Y-M)		[201]
	BG	50	Solid disk	~ 190 (E-M)		[202]
	nBG	30	8 ± 5 vol %	383 ± 50 (E-M)		[203]
	Calcium phosphate	10	Solid fibers	7.55 ± 0.70 (Y-M)		[204]

E-M: Elastic modulus; T-M: Tensile modulus; C-M: Compressive modulus; Y-M: Young’s modulus; T-S: Tensile strength.

**Figure 3:**
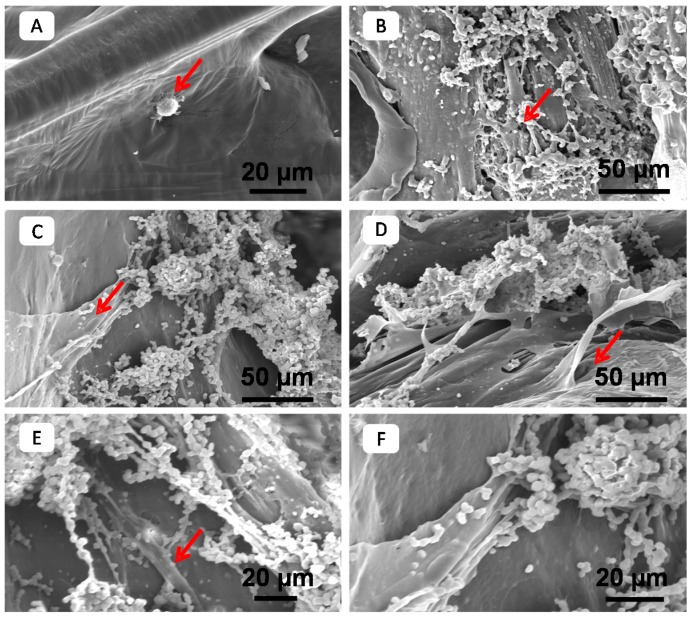
Images of cells cultured on (**a**) PCL scaffold; and (**b**–**f**) PCL/elastin composites. Top surfaces are shown in (**a**) and (**c**), cross sections in (**b**) and (**d**–**f**), arrowheads in the images show representative cells 50 mg/mL elastin solution was used to form composites. Figure reproduced from [209], with permission from Elsevier.

Natural-based fillers such as alginate, chitosan, gelatin, collagen and eggshell powder were used to improve the cell compatibility and hydrophilicity of PCL [193,194,195,196,205,206,207]. For instance, the addition of 10 wt % alginate resulted in an eight-fold enhancement in water absorption, 1.6-fold enhancement of cell viability at seven days, ~2.3-fold enhancement of ALP activity at 14 days and~6.4-fold enhancement of calcium mineralization at 14 days. In addition, chitosan-PCL composite supported neuron-like PC-12 cell adhesion and showed a significantly higher β-tubulin gene expression. A composite of gelatin, chitosan and PCL were used for cardiac tissue engineering. This proposed cardiac patch had a sufficient mechanical strength along with allowing migration or pre-loading of cardiac cells in a biomimetic environment. Collagen type I was also coated on the surface of PCL and PCL-gelatin composite for skin tissue engineering and wound healing applications. The optimum adhesion, viability and proliferation of L929 fibroblast cells on the surface of the composite were observed after surface modification with 1 wt % collagen type I. In another study, a semi-interpenetrating polymer network structure of PCL and elastin was prepared. In this approach, we initially fabricated a porous structure of PCL by using a gas foaming technique. Subsequently, elastin was impregnated within the structure of PCL under high-pressure CO_2_ and crosslinked *in situ* as it can be seen in Figure 3. *In vitro* studies with chondrocyte showed that the incorporation of elastin within the structure of PCL enhances cell proliferation and adhesion, [208,209]. Therefore, these scaffolds may be suitable for cartilage tissue regeneration.

The composites of PCL with inorganic/organic compounds such as graphene, multiwall carbon nanotubes (MWCNTs), PEG, PLA and PU have been prepared to enhance its mechanical properties [197,198,210,211,212,213]. The graphene and MWCNTs were mainly used for electro-responsive tissue types and improvement of mechanical performances. However, adverse effect on cell viability and proliferation was observed when using graphene and MWCNTs above 1 and 0.5 wt %, respectively. A 3D scaffold made of PCL and 30 wt % HA was designed by Shor *et al.* with improved mechanical properties and enhanced bioactivity [214]. The melt blending method was used for the fabrication of PCL/HA composites, and precision extrusion deposition system was developed at Drexel University to fabricate a scaffold with porosities from 60% to 70% and pore sizes from 450 to 750 µm. Another study was used to investigate the feasibility of producing highly porous PCL/BG composite via solid-liquid phase separation method for bone tissue engineering [215]. A porous scaffold with the porosity of 88%–92% and the highest elastic modulus of 251 ± 32 kPa was constructed using either dimethyl carbonate or dioxane as a solvent, and ethanol as an extracting medium. Additionally, the *in vitro* mineralization in SBF solution four weeks post incubation showed the role of BG particles in the development of apatite.

More recently, a 56-week experiment was conducted to assess the effect of degradation of PCL and its composite after the addition of 5 wt % bioactive glass on the pH of the media [201]. After a sudden increase to 8.36 in pH after the first week of the composite, the pH decreased; however, the pH of the pure PCL medium remained acidic with a drop from 6.5 to 5.1 until eight weeks. The pH values for all the samples slowly increased and ultimately approached a plateau; near 6 for PCL and 8.3 for the composite after the 14th week. The results underlined that the addition of ceramic fillers can eventually neutralize the acidic degradation of polyesters; however, there is no guarantee to keeping the pH neutral which is favorable for cell response.

**Figure 4:**
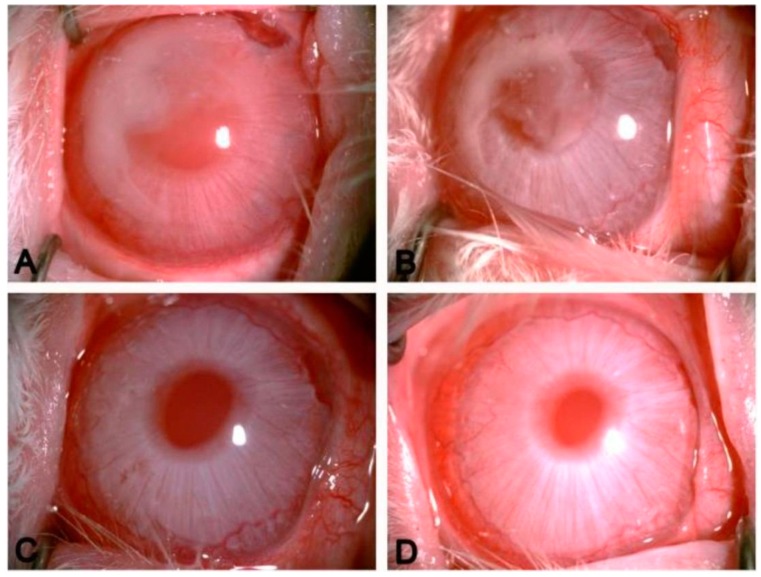
*In vivo* gel formation of PECE hydrogel in the anterior chamber of rabbit. PECE was absorbed completely within three weeks. (**A**) 1 day after injection; (**B**) 7 days after injection; (**C**) 14 days after injection; (**D**) 21 days after injection (×40 magnification) [227].

Similar to all the other polyesters, there has been a major shift towards the chemical modification of PCL to finely tune the physicochemical properties of the polymer. The chemical copolymerization of caprolactone with functionalized monomers such as lactide [216], ethylene glycol [217,218,219,220], monomethyoxy poly(ethylene glycol) [221], acryloxy [222,223,224], and propylene fumarate [225] is used to form a new class of PCL-based polymers. In these chemical modification approaches, the ring opening polymerization technique is used to copolymerize the building monomer of PCL (caprolactone) with different monomers to ultimately alter the physicochemical properties of the resulting polymers. For instance, the multi-block copolymerization of PCL and PEG introduce the thermo-sensitive hydrogel with a promising gel strength and a controllable degradation profile [226]. Interestingly, the sequence of the constructive blocks has a significant impact on the mechanical properties and degradation profile of these copolymers [226]. A block copolymerization of mPEG and PCL was another example of an injectable hydrogel with proper gel strength [221]. Furthermore, an ocular delivery implant was recently developed by Peng *et al.* based on a PEG-PCL-PEG copolymer [227]. The thermo-responsive injectable hydrogel, loaded with bevacizumal, displayed neither corneal abnormalities nor any other ocular tissue damage, and was absorbed completely after three weeks as it is shown in Figure 4. Furthermore, Suen *et al.* has developed a block copolymer of PEG and PCL nanoparticles loaded with triamcinolone acetonide by nano precipitation to treat age-related macular degeneration [228]. The drug was successfully released from the nano career for up to four weeks at a pH of 7.4. This nano-based drug delivery vehicle shows promising results to replace the current intravitreal injection treatment.

Post-polymerization can be also conducted in order to modify biodegradable polyesters chemically. To this end, abstraction of protons from the polyester by treatment with a base, such as lithium diisopropyl amide, followed by subsequent addition of an electrophilic reagent, such as a halogen- or a carbonyl-containing compound, is a feasible method [21]. For instance, different pendant amine [229], hydroxyl, carboxyl groups [230], and peptides [231] have been used to functionalize the PCL backbone. Hu *et al.* utilized a chemical vapor deposition polymerization technique to functionalize the surface of PCL by poly[(4-amino-*p*-xylylene)-*co*-(p-xylene)]. The functionalized surface was coated by biotin to enhance the cell proliferation on the surface of PCL that resulted in 10-fold higher fibroblast cell ingrowth on the surface of scaffold [229].

PCL is deemed to have the highest potential among polyesters for the development of novel, commercial medical devices. This potential is attributed to the unique physicochemical properties of PCL, the relatively biologically benign biodegradation behavior of this polymer and the possibility for fine-tuning and making extensive chemical modifications.

### 4.6. PPF

Poly(propylene fumarate) (PPF) is a crosslinkable polyester with a wide application in *in situ* tissue engineering [232,233,234]. The presence of unsaturated carbon–carbon bonds in the backbone of PPF provides a unique property to form a crosslinked structure [235]. Despite the fabrication of self-crosslinked PPF [236,237], a variety of injectable solutions of PPF-based networks have been developed in the presence of poly(ethylene glycol)-dimethacrylate [238], PPF-diacrylate [239,240,241], and diethyl fumarate [242] as a crosslinking agent. The physicochemical properties and mechanical strength of the crosslinked PPF networks are predominantly dependent on the molecular weight and the polydispersity of PPF [243], the molecular characteristics of the crosslinking agent [244,245], and the ratio of the constituent materials [246]. Accordingly, different biodegradable scaffolds with an extensive range of properties were fabricated for specific applications including bone [247], ear [248], and nerve [249] tissue engineering.

In line with other polymers, the design of monomeric units is a standard approach for modifying the material characteristics of PPF. For instance, different synthetic and naturally driven macromers were incorporated into the propylene fumarate units to extend its biomedical application. The biosynthetic hydrogel, for example, was developed from alginate-PPF copolymer to form a biocompatible scaffold for cardiac tissue engineering [250,251]. Synthetic macromers such as polyethylene glycol (PEG) [252,253,254,255,256] and polyhedral oligomeric silsesquioxane [257], are also copolymerized with PPF to enhance their mechanical properties as well as promoting their biological performance.

## 5. Conclusions

Polyesters are biocompatible and biodegradable polymers that are broadly used for different medical applications as inert medical meshes, physical fixation supports or drug delivery vehicles. To extend the application of these polyesters to regenerative medicine and tissue engineering, it is necessary to modify them to acquire more hydrophilic and cell-interactive polymers. To this end, a series of physical and chemical modification approaches to different polyesters have been used. Among all polyesters, it is deemed that PLA and PCL have the highest potential for future application in medical devices due to their unique physicochemical properties. In addition, the commercial application of PPC and PHB may also be driven by environmental concerns as these two polymers are synthesized from renewable sources. Furthermore, chemical modification of polyesters is considered more favorable than physical modification as it can be scaled up in a more reproducible manner. Different modifications of polyesters in the future may lead to the production of a novel class of polymers on a commercial scale that are more processable, soluble in aqueous based solutions, more biologically active and display variable physicochemical properties.

## Abbreviations

The following abbreviations are used in this manuscript:

PLA: Poly(lactic acid)
PLGA: Poly(lactic-*co*-glycolic acid)
PCL: Poly(ε-caprolactone)
PHB: Poly(3-hydroxybutyrate) or Poly(β-hydroxybutyric acid)
PHBV: Poly(3-hydroxybutyrate-*co*-3-hydroxyvalerate)
PPC: Poly(propylene carbonate)
PBS: Poly(butylene succinate)
PPF: Poly(propylene fumarate)
TPU: Thermoplastic polyurethane
Y-M: Young’s modulus
T-S: Tensile strength
C-S: compressive strength
R: resistance
E-M: Elastic modulus
S: stiffness
T-M: Tensile modulus
C-M: Compressive modulus
S-M: storage modulus
PHAs: Polyhydroxyalkanoates
PEO: Polyethylene oxide
PEGM: Polyethylene glycol methacrylate
CO_2_: Carbon dioxide
GO: Graphene oxide
NIPU: Non-isocyanate polyurethane
HA: Hydroxyapatite
TCP: β-tricalcium phosphates
MWCNTs: Multiwall carbon nanotubes
BG: Bioglass
PLEOF: Poly(lactide-ethylene oxide fumarate)
HEMA: Hydroxyethyl methacrylate
PEG: Polyethylene glycol
